# Acknowledgment of Reviewers

**DOI:** 10.3390/nu4030151

**Published:** 2012-02-23

**Authors:** Peter Howe

**Affiliations:** *Editor-in-Chief of Nutrients*, Nutritional Physiology Research Centre, Sansom Institute for Health Research, School of Health Sciences, University of South Australia, Adelaide, South Australia 5001, Australia; E-Mail: peter.howe@unisa.edu.au; Tel.: +61-8-8302-1200; Fax: +61-8-8302-2178

It is with great appreciation that we acknowledge the following reviewers who have served our research community by reviewing manuscripts for *Nutrients* in 2011.

**Table:** 

Stuart L. Abramson	Anette E. Buyken	Zora Djuric
Bharat Aggarwal	Philip Calder	Fernanda Domingues
Samuel E. Aggrey	Carlos Camargo	Marjorie Ellin Doyle
Said Ajlouni	John J. Cannell	Gregg Duester
Juan Pablo Albar	Francesco Cantatore	Erik Eckhardt
Michael Amling	Christelle Cebo	Garry Egger
Amy Anderson	Hyung-Kyoon Choi	Susumu Eguchi
John J. B. Andersson	Sara Cicerale	Hani S. El-Nezami
Pinarosa Avato	Milan Ciz	Lisa Elviri
Giorgio Barbarini	Karine Clément	Peter Erickson
Alan Barclay	Robert Cooney	François Fenaille
Susan I. Barr	Anthony Corfield	Stefano Fiorucci
Sabine Baumgartner	Robert Cousins	Julie Fisher
Craig R. Baumrucker	Georgina Crichton	Lynda A. Frassetto
Christopher Bell	Francesca L. Crowe	William T. Gibson
Ina Bergheim	Adrian G. Cummins	Sandra Godden
Marla J. Berry	Susanna Cunningham-Rundles	Lourdes Gómez-gómez
Elizabeth R. Bertone-Johnson	Geraldine Cuskelly	Michael D. Griswold
Christophe Blecker	Teresa Lopes Da Silva	Conny Gysemans
Brian C. Bowker	Ralph W. De Vere White	Susan J. Hagen
George Bray	Eric A. Decker	Charles Halsted
Thomas Brenna	Hector Deluca	Akimasa Hatanaka
Lindsay Brown	Ulrich Desselberger	Robert P. Heaney
David Heber	Lynne V. Mcfarland	Valeria Sibilia
Jaimie Hemsworth	Samia Mezouari	Gilbert H. Smith
Paul Hewlett	Carla K. Miller	Mario Soares
Allan G. Hill	Michelle Mitchell	Danfeng Song
Chi-Tang Ho	Antonio Morandi	Eliana B. Souto
Jonathan M. Hodgson	Lee E. Morrow	Ivan Spasojević
Michael Holick	Mark Moss	Gregory T. Spear
Licia Iacoviello	Jorge Mujico	Bonnie Specker
Elena Ibañez	Kazutoshi Nakamura	Lyn M. Steffen
Masahiko Ikeda	Masanobu Nanno	Francene M. Steinberg
Sin-hyeog Im	David C. Nieman	Kerst Stelwagen
Filip Van Immerseel	Cristina Novembrino	Poul Suadicani
Philippa Jackson	Rima Obeid	Russell Swerdlow
David M. Janicke	William T. Oliver	Michael Symonds
Richard J. Johnson	Daniel J. O’sullivan	Shahrad Taheri
Kieran Jordan	Casey M. Owens	Francis A. K. Tayie
Nathalie Juge	Jeetesh Patel	Carla Taylor
Taiho Kambe	Simon H. S. Pearce	Masaaki Terashima
Leila J. Karhunen	Francisco J. Pérez-Cano	Reiko Teshima
Takeshi Katayama	Vincenzo Pezzi	David Thomas
Joseph Katz	Eduardo Pimenta	Diana Thomas
Shannon L. Kelleher	Lindsay D. Plank	Kieran M. Tuohy
Jae Cheol Kim	Anna Poli	Cuno S. P. M. Uiterwaal
Todd R. Klaenhammer	Loredana Quadro	Zeynep Ustunol
Kimberly Kline	William D. Rees	Patrícia Valentao
Koichi S. Kobayashi	Manja Reimann	Luca Vangelista
Joanne Kotsopoulos	Graziano Riccioni	Sandra G. Velleman
Laurie L. Lachance	Amy Riek	Pietro Vernazza
Carl J. Lavie	Ger T. Rijkers	Peter Vestergaard
Derek LeRoith	Joseph F. Robare	Michael H. Walter
Selma C. Liberato	Cécile Rochette-Egly	Connie Weaver
Ausgusto Litonjua	David S. Rosenthal	Eric C. Westman
Kerry M. Loomes	Ryuji Sakakibara	Susan J. Whiting
Alfredo J. Lucendo	Francisco J. Sánchez-Muniz	Martin G. Wilkinson
Faidon Magkos	Robert B. Saper	David S. Wishart
Philippe Marteau	Peter Schemmer	Matthias Wjst
Peter Mccaffery	Lutz Schomburg	Richard Wood
Craig McClain	Michael Schubert	Kun Zhu
Gordon J. Mcdougall	Ulrich Schweizer	Karin Zitterl-Eglseer
Aidan Mcelduff	Je Kyung Seong	

I also wish to draw your attention on the *Nutrients* website to our Editorial Board and also to the guest editors of our expanding list of special issues who have devoted their time and expertise to achieving rapid growth in the quantity and quality of published manuscripts. My special thanks also to Associate Editor, Jon Buckley, and members of the editorial office for their dedication to the journal.

